# Supplementary material for: A Tale of Four Danish Cities: Legionella pneumophila Diversity in Domestic Hot Water and Spatial Variations in Disease Incidence
Source: Int J Environ Res Public Health. 2022 Feb 22;19(5):2530. doi: 10.3390/ijerph19052530 (PMC8909801; doi:10.3390/ijerph19052530)
Supplement: Supplementary file 1 [file ijerph-19-02530-s001.zip › ijerph-1588731-supplementary.pdf]

Supplementary Material

Title: A tale of Four Danish Cities: *Legionella pneumophila* Diversity in Domestic Hot Water and Spatial Variations in Disease Incidence

Supplementary Methods

qPCR Methods:
A half-liter of each sample was processed with the Extraction Pack Environment 03 and GeneDisc Ultra-Lyser according to the manufacturer's instructions (Pall GeneDisc Technologies, New York, USA). The DNA was extracted in a volume of 500 µl and 5 µl was investigated by qPCR detecting *Legionella* ssp. *Legionella* (*L.*) *pneumophila* and *L. pneumophila* serogroup (SG) 1 in separate wells. In-house primers and probe were used for detecting a sequence of *Legionella* spp. 16S DNA. The primers and probes for *L. pneumophila* (*mip*) and LP SG 1 (*wzm*) were according to Mentasti et al., 2015 [1]. An in-house DNA standard (LP SG1 subgroup Knoxville) was calibrated with the *L. pneumophila* Standard DNA EGDUP1 (Pall GeneDisc Technologies, New York, USA). Results are presented as Genome Units per liter (GU/L). Results for *Legionella* spp. qPCR is not presented and results for qPCR for *L. pneumophila* and SG 1 are only presented as a summary. The limit of detection for the method applied on half-liter samples is 500 GU/L.

Supplementary Results

qPCR Results
qPCR for *Legionella* spp., *L. pneumophila,* and *L. pneumophila* SG1 were performed on samples from every second system (valid results from 44 systems, 88 samples). The aim was to evaluate the method compared to culture. The main finding was a fairly good correlation between *L. pneumophila* qPCR and culture (R2=0.6). All but one (with 200 CFU/L) *L. pneumophila* culture-positive samples (n=60) were also positive by *L. pneumophila* qPCR (98%). Of 20 culture-negative samples, 13 were also *L. pneumophila* qPCR negative (65%), for the remaining qPCR had values below 1,000 GU/L for five samples (25%), and between 2,000 and 5,000 GU/L for two samples. In 23 samples with >10,000 LP CFU/L, *L. pneumophila* qPCR had values of >10,000 GU/L for 22 samples (96%), one sample had 4,000 GU/L (probably due to partial inhibition). qPCR was performed for five systems positive for SG1 by culture. SG1 was detected by qPCR for samples from four of the systems, in one system qPCR confirmed that the system in City B was colonized by SG1 only. Samples from one system were negative for SG1 by qPCR but the SG1 concentration was probably below the detection limit for qPCR as only one SG1 colony was recovered from the Flush sample and the system was predominantly colonized by *L. pneumophila* SG3. Two systems in City B were negative for SG1 by culture but qPCR positive, both systems were predominantly colonized by *L. pneumophila* non-SG1 and *Legionella* spp. (*L. anisa* and *L. londiniensis* respectively) and SG1 colonies could have been overlooked as only few colonies (six from each system) were investigated by the Oxoid kit. None of the qPCR investigated samples from City C and D were positive for SG1. All investigated samples were positive for *Legionella* spp.

Supplementary Discussion

Discussion of qPCR
It is well known that culture and qPCR are not equivalent methods, qPCR will generally overestimate the number of cultivable *Legionella* especially for *Legionella* non-*pneumophila*. In addition, qPCR can detect dead and viable but nonculturable (VBNC) *Legionella*. However, this study among others [2,3] confirms that if *L. pneumophila* qPCR is negative the level of cultivable *L. pneumophila* is also low or negative (the limit of detection for qPCR could be improved by further concentration of the samples). High values obtained by

L. pneumophila qPCR also indicate a high level of cultivable *L. pneumophila* (especially for systems not treated with a biocide or thermal disinfection [3]). If SG1 detection is added to the qPCR, SG1 colonization can be detected and the level estimated much faster than with the culture method. This is especially relevant in an outbreak situation (the vast majority of outbreaks are caused by SG1) where several potential sources must be screened. In this study, qPCR confirms a relative high SG1 colonization rate in City B and low rates in Cities C and D. In addition, SG1 can well be overlooked by the culture method as SG1 often are found in co-culture with *L. pneumophila* non-SG1 or other *Legionella* species and often in higher numbers than SG1.

References

1. Mentasti, M.; Kese, D.; Echahidi, F.; Uldum, S.A.; Afshar, B.; Davis, S.; Mrazek, J.; De Mendonca, R.; Harrison, T.H.; Chalker, V.J. Design and validation of a qPCR assay for accurate detection and initial serogrouping of Legionella pneumophila in clinical specimens by the ESCMID Study Group for Legionella Infections (ESGLI). *Eur J Clin Microbiol Infect Dis* **2015** *34*,1387-1393, doi: 10.1007/s10096-015-2363-4.
2. Collins, S.; Jorgensen, F.; Willis, C.; Walker, J. Real-time PCR to supplement gold-standard culture-based detection of Legionella in environmental samples. *J Appl Microbiol* **2015** *119*, 1158-1169, doi:10.1111/jam.12911.
3. Lee, J.V.; Lai, S.; Exner, M.; Lenz, J.; Gaia, V.; Casati. S.; Hatemann, P.; Lück, C.; Pangon, B.; Ricci. M.L.; Scaturro, S.; et al. An international trail of quantitative PCR for monitoring Legionella in artificial water systems. *J Appl Microbiol* **2011** *110*, 1032-1044, doi: 10.1111/j.1365-2672.2011.04957.x.

Supplementary Figures

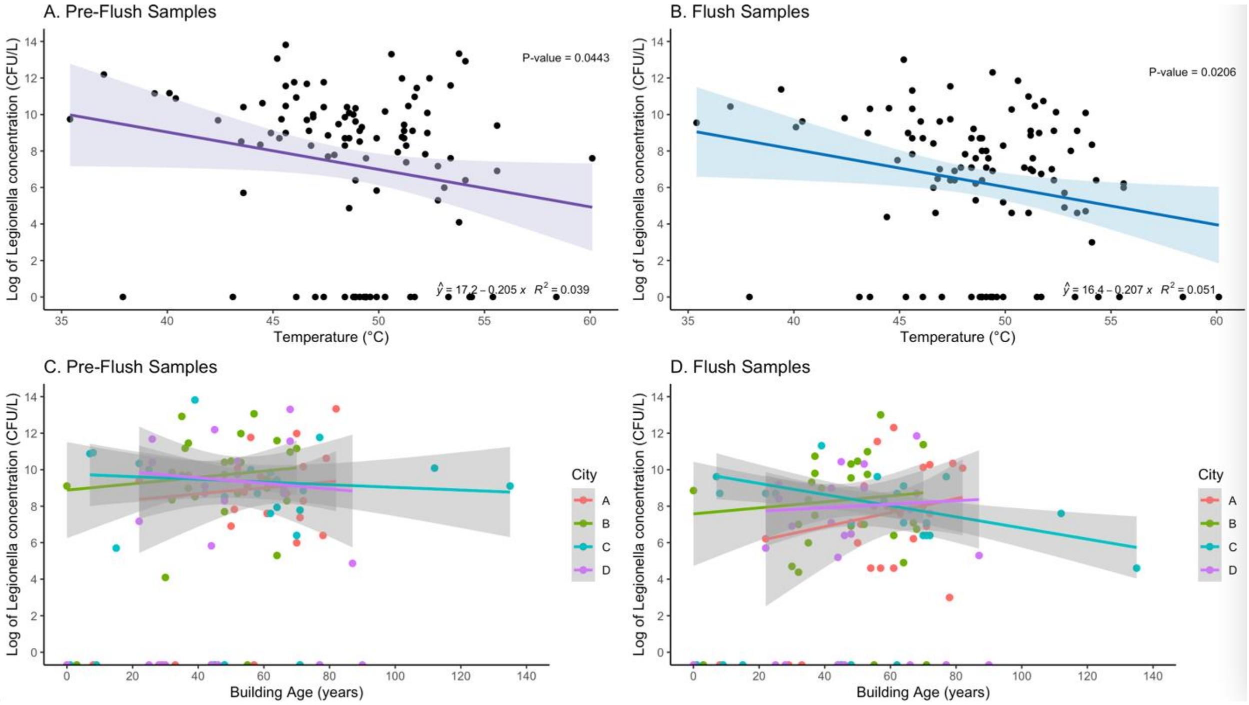

Figure S1. The association between log of *Legionella* concentration (CFU/L) and Temperature (°C) (Panel A and B) and Building Age (years) (Panels C and D) with line of the best fit (linear regression) and R-squared shown for Panel A and B. Including samples that did not detect *Legionella*.

Due to the overdispersion of *Legionella* concentrations (CFU/L), linear models were fit to the log of CFU/L to estimate the association between building level characteristics and *Legionella* concentration. Negative Binomial models were assessed as an alternative and fit to the non-transformed CFU/L data but we did not feel that the difference between a single increase in colony count was environmentally or clinically as relevant as a log increase in colony count.

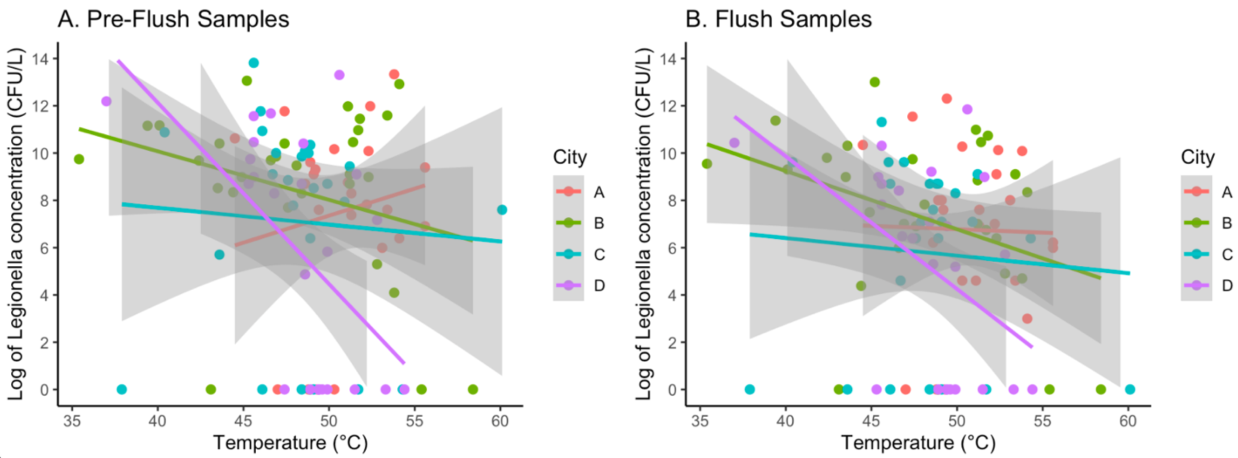

Figure S2. The association between log of *Legionella* concentration (CFU/L) for Pre-Flush and Flush sample and Temperature (°C) of each City.

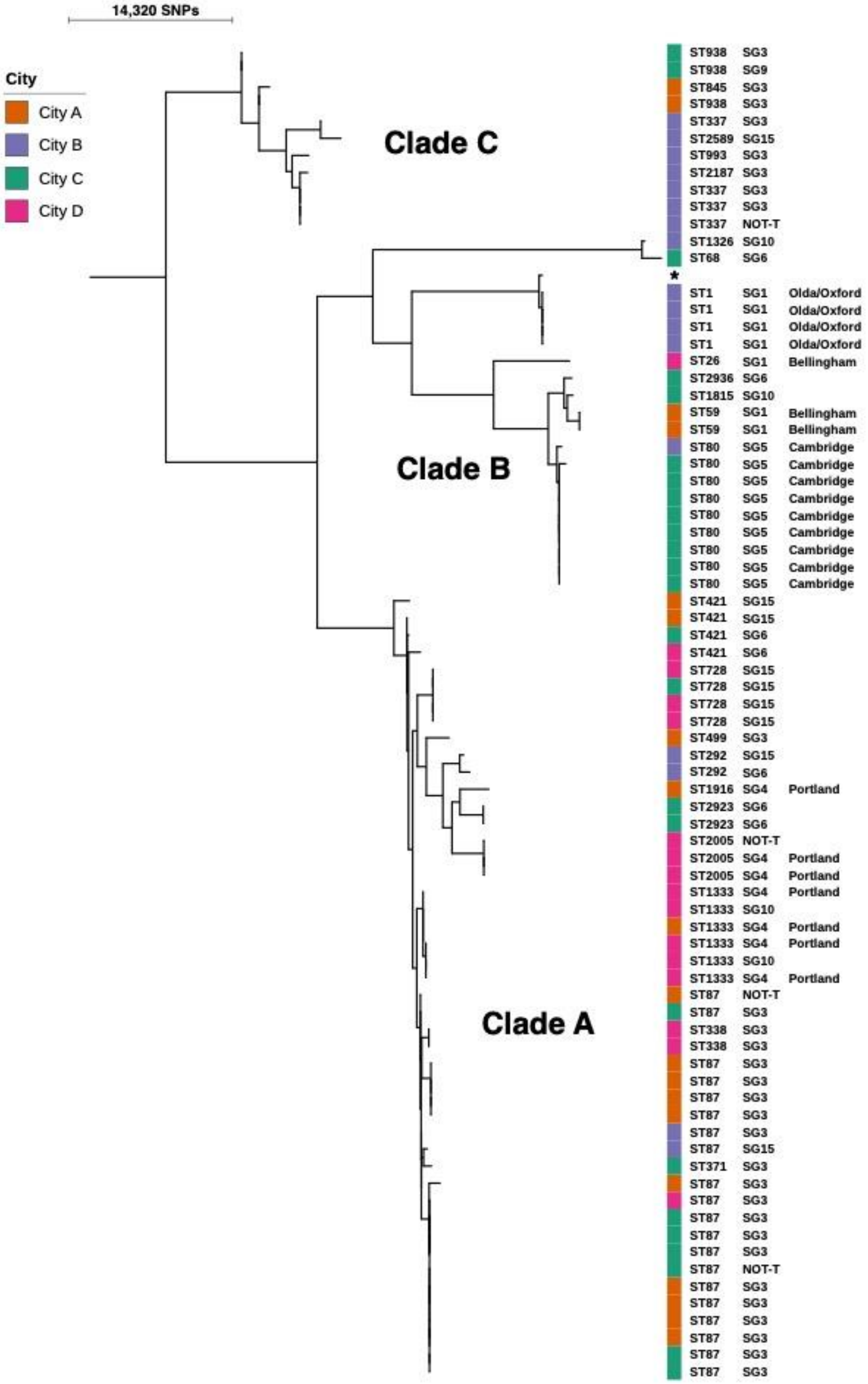

Figure S3. Single nucleotide polymorphism (SNP) analysis containing recombinant regions (unpurged phylogeny) of 77 *L. pneumophila* isolates from the four cities. * Reference, L.pneumophila strain Paris (ST1).

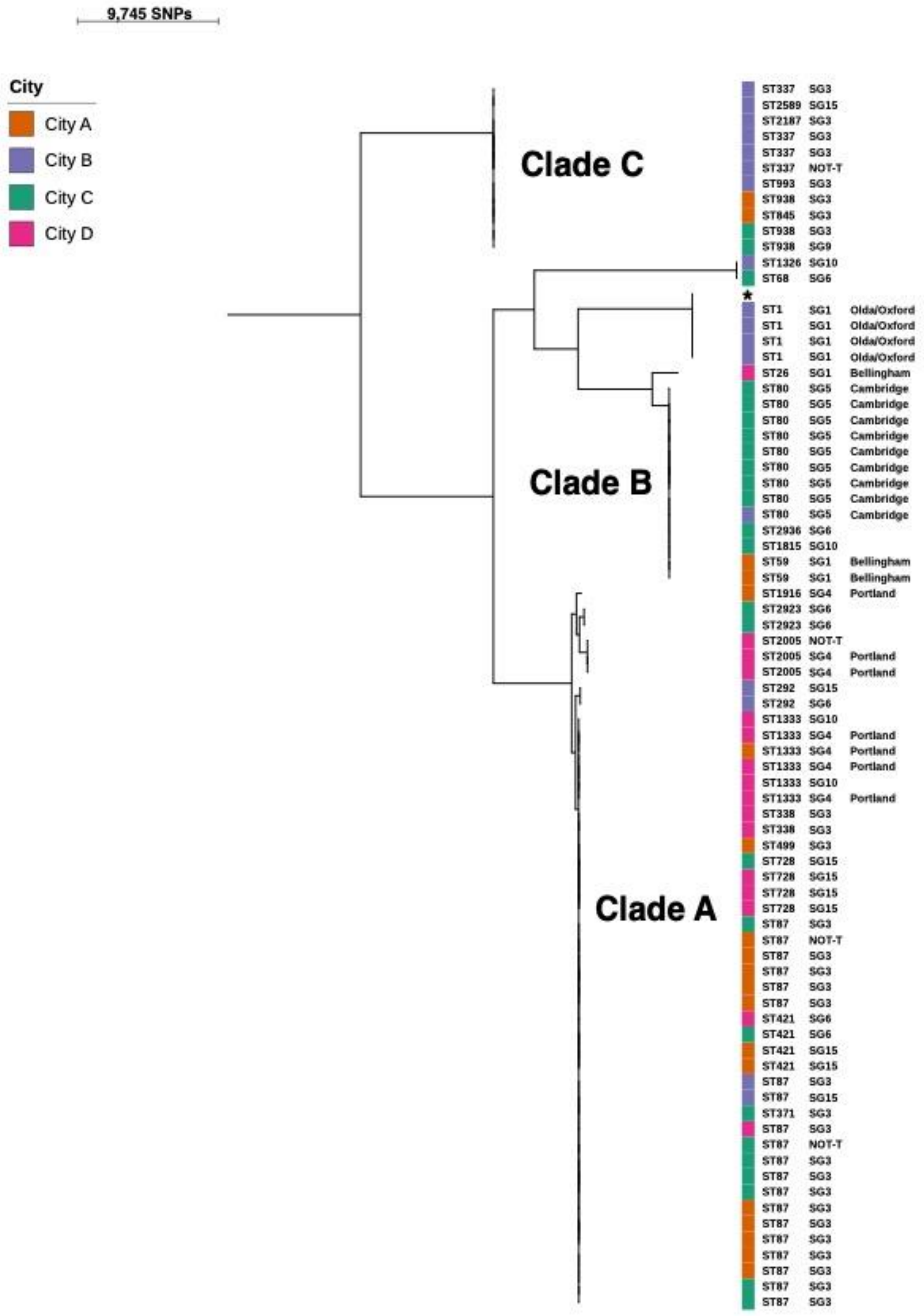

Figure S4. Single nucleotide polymorphism (SNP) analysis without recombinant regions (purged phylogeny) of 77 *L. pneumophila* isolates from the four cities. * Reference, L.pneumophila strain Paris (ST1).
